# mirTarRnaSeq: An R/Bioconductor Statistical Package for miRNA-mRNA Target Identification and Interaction Analysis

**DOI:** 10.1186/s12864-022-08558-w

**Published:** 2022-06-13

**Authors:** Mercedeh Movassagh, Sarah U. Morton, Christine Hehnly, Jasmine Smith, Trang T. Doan, Rafael Irizarry, James R. Broach, Steven J. Schiff, Jeffrey A. Bailey, Joseph N. Paulson

**Affiliations:** 1 Dana Farber Cancer Institute and Harvard T.H. Chan School of Public Health, Boston, MA United States; 2 Boston Children’s Hospital and Harvard Medical School, Boston, MA United States; 3 Institute for Personalized Medicine, Department of Biochemistry and Molecular Biology, The Pennsylvania State University College of Medicine, Hershey, PA United States; 4 Center for Neural Engineering and Center for Infectious Disease Dynamics, Departments of Engineering Science and Mechanics, Neurosurgery and Physics, The Pennsylvania State University, University Park, State College, PA 16802 USA; 5 Warren Alpert Medical School, Brown University, Providence, RI USA

## Abstract

We introduce *mirTarRnaSeq*, an R/Bioconductor package for quantitative assessment of miRNA-mRNA relationships within sample cohorts. *mirTarRnaSeq* is a statistical package to explore predicted or pre-hypothesized miRNA-mRNA relationships following target prediction.

We present two use cases applying *mirTarRnaSeq*. First, to identify miRNA targets, we examined EBV miRNAs for interaction with human and virus transcriptomes of stomach adenocarcinoma. This revealed enrichment of mRNA targets highly expressed in CD105+ endothelial cells, monocytes, CD4+ T cells, NK cells, CD19+ B cells, and CD34 cells. Next, to investigate miRNA-mRNA relationships in SARS-CoV-2 (COVID-19) infection across time, we used paired miRNA and RNA sequenced datasets of SARS-CoV-2 infected lung epithelial cells across three time points (4, 12, and 24 hours post-infection). *mirTarRnaSeq* identified evidence for human miRNAs targeting cytokine signaling and neutrophil regulation immune pathways from 4 to 24 hours after SARS-CoV-2 infection. Confirming the clinical relevance of these predictions, three of the immune specific mRNA-miRNA relationships identified in human lung epithelial cells after SARS-CoV-2 infection were also observed to be differentially expressed in blood from patients with COVID-19. Overall, *mirTarRnaSeq* is a robust tool that can address a wide-range of biological questions providing improved prediction of miRNA-mRNA interactions.

**Supplementary Information:**

The online version contains supplementary material available at 10.1186/s12864-022-08558-w.

## Introduction

Non-coding RNAs (ncRNAs) are important for the maintenance of homeostasis in many organisms [1]. microRNAs (miRNAs) are a class of ncRNAs that are single stranded and 18-22 nucleotide bases in length, which can alter messenger RNA (mRNA) expression [2]. miRNAs, in general, primarily downregulate mRNA expression through translational repression during initiation or elongation, or via mRNA degradation through cleavage by the RNA-induced silencing complex through argonaut (AGO) proteins (more commonly observed in plants, and viruses) or by interfering with PABPC-EIF4G interactions, facilitating translational repression and deadenylation [3–5]. Full or partial matching in the seed region (nucleotide 2-8) of the 5′ end of miRNA with the target mRNAs 3’UTR sequence leads to sequence-specific miRNA-mRNA interactions [6]. However, the presence of potential binding interactions are not sufficient to predict in vivo mRNA regulation by miRNAs as many predicted interactions are not observed in cell-based assays.

miRNAs robustly regulate mRNA expression during developmental or pathological processes such as cell proliferation, metabolism, immunity, and organism development by targeting multiple relevant biological levels/pathways simultaneously [7]. miRNAs also have roles in both host response to infection and viral suppression of the host immune response, as has been recently exemplified by Severe acute respiratory syndrome coronavirus 2 (SARS-CoV-2/COVID-19) [8–10]. Notably many viruses, in particular herpes viruses, express viral miRNAs to regulate both the host and viral mRNA expression [11]. For example, Epstein Barr Virus (EBV), the causal agent for mononucleosis, is associated with multiple cancers which express high levels of EBV miRNA and mRNA [12, 13]. These miRNAs regulate immunological processes in the host such as the WNT signaling pathway, interleukin (IL) inflammatory response or promote cell growth or inhibit apoptosis in various cancers [12].

There are several computational algorithms which predict the targets of human and viral miRNAs. Algorithms such as *miRanda*, *TargetScan* or *VIRmiRNA* database (db) generally predict miRNA-mRNA interactions based on 6-8mer seed matching, free energy estimated for each miRNA-mRNA target pair, evolutionary conservation of the 3′ UTR sequence. In the case for *miRanda* in general a predicted target can be ranked high (high score) in the results by either obtaining a high individual score or by having multiple predicted sites [13]. *TargetScan* ranks predicted targets by either targeting efficacy or the probability or conservation [14]. Tools such as *VIRmiRNA* db, report on either experimentally verified miRNA-mRNA interactions or primarily reports on miRNA seed conservation amongst viral and cellular miRNAs [15]. *FilTar* and other modeling methods have been used successfully for predicting potential miRNAs-mRNA targeting [16–18]. However, these tools are mainly presented as online databases and designed for host or viral miRNAs prediction of potential mRNA targets limited to specific organisms [19]. There is a need for computational packages to assess downstream miRNA-mRNA predictions leveraging the expression data from high throughput sequencing analyses of experimental data in a statistical framework while testing or accounting for various phenotypes of interest of eukaryotic and viral miRNAs.

Here, we have developed *mirTarRnaSeq*, an R/Bioconductor package which can measure statistical relationships between miRNAs and mRNAs. Our methods are split into three parts. Part 1: Regression analysis (univariate and multivariate modeling). The main question we aim to answer in this section is, if there is statistical evidence for miRNA-mRNA relationship across the matched cohort. Part 2: Correlation and sparse partial correlation analysis. Assessing the correlation between miRNAs and mRNAs across three or more time points. Part 3: Differential fold-change analysis. Assessing the miRNA and mRNA interactions at two time points. Pre-processed miRanda miRNA-mRNA prediction files for four species (human, mouse, fly and Caebirhabditis elegans (c-elegans)) and three viruses (EBV, human cytomegalovirus, and Kaposi sarcoma herpes virus) are included in the package; users can supply mirRanda data (or any other miRNA-mRNA prediction method compatible with *mirTarRnaSeq*) for additional organisms of interest to be able to use *mirTarRnaSeq*. As a proof of concept, we applied *mirTarRnaSeq* to three datasets demonstrating the utility and providing novel results for gastric adenocarcinoma and SARS-CoV-2. The first dataset contains 25 matching miRNA and mRNA stomach adenocarcinoma (STAD) samples from The Cancer Genome Atlas (TCGA) reported to have highly-expressed EBV miRNAs [20]. For Part 2 and 3 we leverage paired mRNA and miRNA libraries from lung epithelial cells sampled at 4, 12 and 24 hours after infection with SARS-CoV-2. We report miRNA-mRNA regulation during viral infection, both previously established as well as novel interactions. We identify miRNA-mRNA interactions during host response to EBV infection in stomach adenocarcinoma and predict EBV self-regulation by viral miRNAs. Further, we identify miRNAs predicted to regulate targets involving interleukin and interferon pathways during the host response to SARS-CoV-2. We further validate the miRNA-mRNA interactions in Part 3 by comparing a dataset of four acute COVID-19 infections as compared to four control individual blood samples and find convergence in miRNA-mRNA targeting between the two experiments. Here we demonstrate the utility of tools detecting both previously validated interactions as well as potential novel ones including host response pathways in EBV-positive stomach adenocarcinoma and inflammatory response in interleukin and interferon pathways in the context of SARS-CoV-2 infection.

## Materials and Methods

### Host-virus interactions (Part 1. regression)

#### Sample selection and analysis

Matching miRNA and mRNA samples for 50 tumors were downloaded from The Cancer Genome Atlas (25 EBV positive and 25 EBV negative) (TCGA, http://cancergenome.nih.gov/) accessed through the Cancer Genomic Hub (https://gdc.cancer.gov/) (Supplemental Table S1) [20]. Currently, TCGA does not provide transcript information for viral miRNA and mRNAs, hence for consistency with previous work, we estimated the number of viral transcripts in the dataset using the pipeline described previously [21].

#### Viral miRNA and mRNA detection

TCGA identifiers for samples with high and no expressed EBV miRNAs are listed in Supplemental Table S1. Downloaded binary alignment map (bam) files were converted to fastq files using *bedtools* bam2fastq (V2.29.2) [22]. Concatenated EBV reference (NC_007605) and human (hg19) genome were used to detect best placement for viral miRNA as previously described [21]. The miRNA alignment was done with recommended miRNA alignment parameters; zero mismatch for 8 base pair seeds using *Burrows-Wheeler Aligner* (*bwa*-0.7.17). To remove human contaminated cross mapping of viral and human miRNAs, we extracted uniquely aligned miRNAs using *samtools* (V0.1.19), to EBV (NC_007605) reference genome. miRNA fragments were then quantified based on their defined genomic locations (*miRBase* version 22) as counts per million (CPM) using *bedtools* and in-house parsing scripts [22, 23]. For consistency with previous methods on detecting EBV transcripts, for EBV mRNA alignment *TopHat2* [24] was used and for viral miRNA alignment *Burrows-Wheeler Aligner* (*bwa*-0.7.17) was utilized. mRNA fragments were extracted as reads per million (RPM) using *bedtools* and in-house parsing scripts [22, 23]. For human mRNA, fastq files were aligned to the hg19 (GRCh37.p13) reference genome with *TopHat2* using default settings [24]. Gene expression was quantified using the *Cufflinks* (V2.2.0) using default settings and the transcript annotation was obtained from *Gencode*. The assembled transcripts were merged using *Cuffmerge* (V2.2.1) and differential expression was performed using *Cuffdiff* (V2.2.1) to identify the differentially expressed genes between the high and unexpressed EBV miRNA samples [24, 25].

### Differential interactions of mRNA-miRNA predictions across groups or time (Part 2 and Part 3): sample selection and analysis

miRNA and mRNA counts in dataset GSE148729 from human lung epithelial cells infected with SARS-CoV-2 or uninfected control cells were download from Gene Expression Omnibus at NCBI (https://www.ncbi.nlm.nih.gov/geo). The dataset included technical duplicates, resulting in a total of 12 SARS-CoV-2 infected and 12 uninfected samples. We performed differential expression analysis with *DESeq2* [26]. For Part 2, differential expression for mRNA and miRNAs were determined for SARS-CoV-2 compared to uninfected control samples at four hours (h), 12 h and 24 h after infection after removing all genes with at least one count in fewer than two of the six samples. For Part 3, differential expression for mRNA and miRNAs were determined for SARS-CoV-2 infected samples at 4 h versus (vs) 12 h, 4 h vs 24 h, and 12 h vs 24 h after removing all genes with at least one count in fewer than seven of the 12 samples. For assessment of predicted mRNA-miRNA relationships in Part 3 during COVID infection RNA sequencing files were aligned to hg38 reference genome (GRCh38, assembly accession GCA_000001405.27) utilizing *STAR* (2.7.9a) [27]. *RSEM* (v1.3.2) was used for mRNA transcript quantification and *DESeq2* was utilized for differential expression for mRNA [28, 29]. The miRNA alignment was done with recommended miRNA alignment parameters; zero mismatch for eight base pair seeds using *Burrows-Wheeler Aligner* (*bwa-*0.7.17). miRNA fragments were then quantified based on their defined genomic locations (*miRBase* version 22) as counts per million (CPM) using bedtools and in-house parsing scripts (24, 27).

### COVID-19 Sample collection

Confirmatory COVID-19 data was generated through the Penn state PRIDE program. Blood samples had different collection and/or transportation requirements depending upon their research needs and were collected and transported according to PRIDE Program BioRepository standard operating procedures (procedure for EDTA blood and serum attached). Routinely, PRIDE Program blood samples were obtained during clinically ordered blood draws as extra tubes following collection of the clinically ordered tubes. Orders for the extra research tubes were entered as an electronic lab order or ordered manually and contained instructions for the phlebotomist for the collection of additional tubes of blood (less than 30 ml total volume).

### RNA extraction

Blood samples were collected from a total of eight patients (four COVID-19 infection patients and four patients without infection). RNA was extracted from shield samples using TRIzol™ LS Reagent (Invitrogen, 10,296,010) and Direct-zol™ RNA MiniPrep Plus kit (Zymo Research, R2072). Briefly, samples were lysed by adding 750 μl or 1.5 mL of TRIzol™ LS Reagent into 250 μl or 750 μl (RNALater preserved samples) of each sample then extraction was completed following the manufacturer’s protocol with the DNase I treatment. The collected RNA was used for miRNA and mRNA sequencing.

### RNA sequencing

For RNA sequencing, ribosomal RNA was depleted with Ribocop rRNA and Globin Depletion Kit Human/Mouse/Rat (Lexogen, 145) and libraries were prepped with theCORALL Total RNA Library Prep Kit (Lexogen, 119) following the manufacturer’s protocol using 10-150 ng of RNA... The libraries were quantified by size and quality-checked (QC) on an Agilent Technologies 2100 Bioanalyzer. Libraries were then pooled to four nM, diluted and sequenced on the NovaSeq 6000 using the S2 flow cell with 2 × 100 reads aiming for 25 million reads per sample.

### miRNA sequencing

For miRNA sequencing QIAseq® miRNA Library Kit (Qiagen, Germany) was utilized following the manufacturer’s protocol. Briefly, 3′, followed by 5′ ligation was performed on the RNA followed by reverse transcription followed by library prep with amplification. QC and size quantification was performed for all samples on an Agilent Technologies 2100 Bioanalyzer. Libraries were then pooled to four nM and sequenced on the NovaSeq 6000 using the S2 flow cell with 2 × 100 reads aiming for 10 million reads per sample.

### Resource: miRanda target prediction for multiple organisms

As a resource for users, we have processed and provided multiple organisms *miRanda* target predictions included in the package. All miRNA sequence files were obtained from *miRBase* version 22. *miRanda* was used for miRNA target prediction across 3′ UTR or across genome of *Homo sapiens* (hg19), *Mus musculus* (mm10), *Drosophila melanogaster* (dm6), *Caebirhabditis elegans* (c-elegans)(WBcel235), Epstein barr virus (EBV) (NC_007605), Human cytomegalovirus (HCMV) (NC_00627) and Kaposi sarcoma herpes virus (KSHV) (NC_009333.1) build. The final results included total *miRanda* score, folding energy, seed match score, and alignment characteristics score. These results are accessible as organism-specific options though the *mirTarRnaSeq* package. To analyze data from additional organisms, the user can either run their own miRanda prediction or contact the authors for assistance. The actual code for *mirTarRnaSeq* is compatible with any organism as long as the users provide compatible *miRanda* files for their organism, if not already included in the package. Other predictive models of miRNA-mRNA targeting such as *TargetScan* can also be used as input. In this analysis we demonstrate the package using human and EBV miRNAs with matching RNAseq.

### Tools used for statistical analysis and graphical purposes

The scripts for all analyses are available at: https://github.com/DataScienceGenomics/*mirTarRnaSeq*_Paper.git . The included vignettes also provide a clear description of the tool capabilities, inputs, outputs and analyses of each step. All plots were generated using ggplot2. Cell type deconvolution was performed by Gene Expression *Deconvolution Interactive Tool* (*GEDIT*) [30]. Euclidean distance was measured for hierarchical clustering using the *pheatmap* package (v 1.0.12). *vizNetwork* (v 2.0.9) was used for visualization and distance estimation of network plots. “http://biorender.com/” was used for the cover figure’s sequencer image.

## Results

### Overview of mirTarRnaSeq

*mirTarRnaSeq* is an R package for statistical quantitative assessment of miRNA-mRNA expression relationships within the same sample. A user can simply identify if there is enough statistical evidence of the predicted interactions between miRNA-mRNA actually occurring, through flexible *p*-value and adjusted p-value assignment (not constrained to *P <* 0.05) and by using the appropriate model of analysis tailored to their dataset and biological question. A summary of the methods, inputs, and results from *mirTarRnaSeq* is provided in Supplemental Table S2. The investigated relationships between miRNA-mRNA could be predetermined (known or evaluated through other packages) by the user choice or they could be unknown (and evaluated by *mirTarRnaSeq*) across datasets. *mirTarRnaSeq* is also useful for predicting other non-coding RNA (ncRNA) relationships with miRNAs for example in cases where circular RNAs (circRNAs) function as sponges, miRNAs increase can positively correlate with circRNA expression. The flexibility of mirTarRnaSeq for detecting not only negative but positive relationships allows for this type of analysis. *mirTarRnaSeq* requires (inputs) count, count/transcripts per million (C/TPM) or reads per kilobase of transcript per million mapped reads (RPKM) matrix for both miRNA and mRNA samples (if TPM and RPKM are used we suggest using the scale parameter for regression) (Supplemental Table S2).

For time point or phenotypic differential expression (DE) data we recommend using fold change (FC). Therefore any experimental approach that can be used to generate FC data, such as next-generation sequencing or microarrays, can be analyzed in Parts 2 or 3. If there are multiple timepoints for comparison, each interval can be compared pairwise. As an option, based on the organism of interest, the mRNA gene list can be filtered based on the presence of previously reported miRNA-mRNA interactions predicted by miRanda (prediction by miRNA-mRNA folding energy, miRNA-mRNA length of interaction, evolutionary conservation of the miRNA and overall miRanda Scoring). In the current package we have already run and support four eukaryotic organisms (human, mouse, Drosophila, and c-elegans) and three viruses (EBV, HCMV, KSHV) interaction with their own genome and human genome). *mirTarRnaSeq* is compatible with any organism as long as the user provides compatible miRanda / TargetScan predictions. For input file guidance and functionality of each part of the analysis please refer to (Supplemental Table S2).

**Fig. 1:**
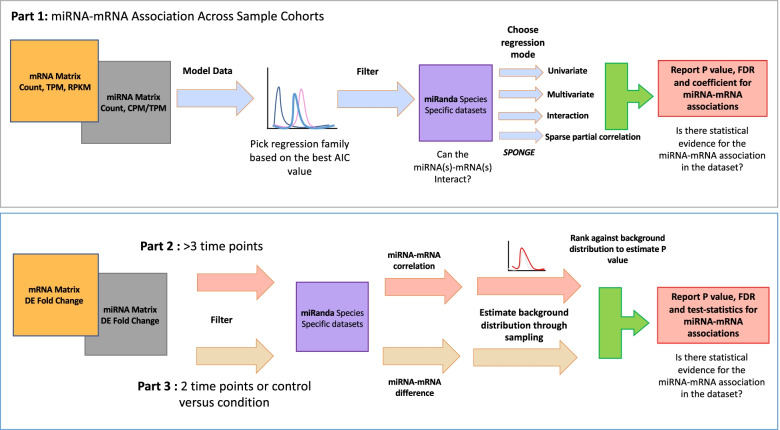
Overall pipeline for mirTarRnaSeq. Part 1: In order to assess miRNA-mRNA relationship across samples, count, transcript/count per million (T/CPM) or read per kilobase per million (RPKM) matrix for miRNA/mRNA sequencing are used as input. The user should do an initial modeling of the data to pick the best regression mode matching their dataset based on the Akaike information criterion (AIC) score. After choosing the appropriate regression model, and organism of interest (for miRanda comparison), the user can now run their model and get a report of the significant miRNA-mRNA relationships to observe if the dataset reflects statistical evidence for the latter relationship. Part 2 (correlation) investigates if there is miRNA-mRNA relationship across time points (T1, T2, T3, ...) or conditions (eg. miRNA-mRNA relationship in high temperature versus cold temperature, verus medium temperature) through correlation. After initial correlation on miRNA-mRNA fold change, a background distribution is estimated through sampling and *P* value is estimated by ranking of the miRNA-mRNA relationship correlation across the background distribution correlation. Part 3 (interrelation) investigates if there is a miRNA-mRNA relationship between two time points (T1 and/or a control and condition. For this we estimate the difference between the miRNA-mRNA fold change. We then form a background distribution for random differences in fold chance and then rank our difference values against the background distribution to get *P* value, FDR and test-statistics estimates

*mirTarRnaSeq* can assess the miRNA-mRNA relationships using several statistical models. These models are either Gaussian, negative binomial, Poisson, zero-inflated Poisson, zero-inflated negative binomial, or the user can choose the multi-model function, where across samples, the best fit model for every miRNA-mRNA relationship is selected. The latter is done through comparison of Akaike information criterion (AIC) for model selection [31]. After determining the appropriate model, and depending on the method of analysis, the package can make statistical predictions and report significant miRNA-mRNA relationships/correlations across miRNA and mRNA datasets (Fig. 1).

To predict miRNA-mRNA relationships, *mirTarRnaSeq* employs four models based on the user’s proposed biological question.

Univariate model Y  =  *β*_0_ + *β*_1_ *χ*_1_  +  *ε*. ⋅

Multivariate model Y  =  *β*_0_ + *β*_1_ *χ*_1_  +  *β*_2_ *χ*_2_  +  *ε*⋅

Interaction/Synergistic model Y  =  *β*_0_  +  *β*_1_ *χ*_1_  +  *β*_2_ *χ*_2_  +  *β*_3_ *χ*_1_ *χ*_2_  +  *ε*⋅

The first method (Part 1) determines the miRNA-mRNA relationship using three types of regression analyses depending on the biological question. Regression analyses can be performed for each transcript. **A.** If the user is interested in univariate relationships between one specific miRNA and one specific mRNA across samples, or relationship of all miRNAs in regards to all mRNAs of interest (one to one relationship across samples) the package will first perform a univariate regression analysis, where Y is the mRNA expression count/CPM/TPM/RPKM (dependent variable), *x*_1_ is the miRNA expression count/CPM/TPM, *β*_1_ is the association of miRNA and mRNA, *β*_0_ is the intercept and *ε* represents the random error for the model. The package then reports on the significant miRNA-mRNA relationships based on the model *p*-values. **B.** If the user is investigating miRNA-mRNA relationships in the presence of another miRNA of interest a multivariate model can be employed, where *β*_2_ *x*_2_ represents the secondary miRNA weight of association with mRNA for the independent variable (miRNA 2). **C.** In the cases where the user is interested in identifying the synergistic relationship between miRNA-mRNAs of interest they can use the interaction mode for their model analysis, where *β*_3_*x*_1_ *x*_2_ is the interaction term and *β*_3_ represents the association of the interaction term with the independent variable (mRNA).

In the multivariate and interaction models of Part 1 *mirTarRnaSeq*, the user can choose to run all miRNA-mRNA relationships; however this option limits the user to investigating relationships two miRNAs at a time. The user can also investigate more than two miRNA-mRNA relationships individually with the option for testing positive or negative miRNA-mRNA relationships (Fig. 1). **D.** Finally, for sparse partial correlation miRNA-mRNA predictions, we have made *mirTarRnaSeq* compatible with the *SPONGE* elastic regularized linear regression model algorithm [32]. We recommend the users to choose this option if they have a large number of samples > 50.

$$r=\frac{\varSigma \left(\ {x}_i-\overline{x}\right)\ \left(\ {y}_i-\bar{y}\ \right)}{\sqrt{\varSigma {\left(\ {x}_i-\overline{x}\right)}^2\ \varSigma {\left(\ {y}_i-\bar{y}\right)}^2}}$$

The second method of analysis, Part 2 (correlation), estimates if there is a significant miRNA-mRNA correlation in three or more time points with or without replicates across samples to estimate the correlation between miRNA and mRNAs of interest across time. The estimated fold-change result of differential expression analysis on miRNA and RNA sequencing is used for analysis. *x* is a vector that contains pair-wise fold change of miRNAs between all time points, and *y* is a vector containing pair-wise fold change of mRNAs between all time points. Where *r* is the correlation coefficient for the relationship between a specific miRNA and specific mRNA across time points in the sample set, *x*_*i*_ is miRNA FC between two time points, $\underset{\_}{x}$ is mean of miRNA fold change across time points, *y*_*i*_ is the mRNA fold-change in one between two time points and $\underset{\_}{y}$ is the mean of mRNA fold-change across time points. We then estimate the *p*-value by comparing *r* to a background null distribution generated by calculating correlations for randomly selected miRNA-mRNA relationships across the sample set (default 100 permutations) (Fig. 1).

$$d\kern0.5em =\kern0.5em \mid \kern0.5em x\kern0.5em -\kern0.5em y$$

The third method (interrelation) (Part 3) is for estimating the miRNA-mRNA interactions between two time points. In this scenario the absolute difference between miRNA-mRNA is *d,* and is estimated between the two points. *x* is the fold change for an individual miRNA and *y* is the fold change for an mRNA. We then estimated p-value by comparing *d* to a randomly selected background distribution of miRNA-mRNA differences across the sample set and ranking (Fig. 1).

### Implementation and availability

*mirTarRnaSeq* is an open source freely available package on Bioconductor DOI: 10.18129/B9.bioc.mirTarRnaSeq. Users can install the package using BiocManager 3.14 or higher:

if (!requireNamespace(“BiocManager”, quietly = TRUE))

install.packages(“BiocManager”)

BiocManager::install(“mirTarRnaSeq”)

The vignette provides an example walk through of the different types of analyses. In short, there are three main biological questions in regards to miRNA-mRNA relationships that mirTarRnaSeq analysis enables the users to answer. The required input for mirTarRnaSeq use is (a) matching miRNA/mRNA sequencing datasets (one miRNA and one mRNA file) and (b) miRNA-mRNA prediction file. The default compatible file for this is a miRanda prediction file, but users may adapt outputs from TargetScan.

For details on the specific biological questions for miRNA-mRNA relationships and details on functions and input files used for every section the vignette, Supplemental Table S2 and Fig. 1 can be accessed.

### Part 1. *mirTarRnaSeq* analysis of 25 samples with high EBV miRNA expression

We applied *mirTarRnaSeq* to TCGA samples from 25 matching miRNA and mRNA sequencing samples from patients with stomach adenocarcinoma with high levels of EBV miRNA expression and 25 patients with no EBV miRNA expression (Supplemental Table S1). EBVGC forms 8-16% of STAD [21]. EBV presence in the epithelial cells of gastric cells has been associated with mucosal damage and the presence of viral genes such as *BARF1* has been shown to promote cell proliferation [33]. It has been shown that in TCGA data ~%8 of gastric adenocarcinoma samples express high levels (> 10^4^ CPM) of EBV miRNAs and 23% show no traces of EBV miRNA or mRNA [21]. We took advantage of the fact that 8% of gastric carcinoma samples found in TCGA have high expression of EBV to analyze this data using *mirTarRnaSeq* and computationally decipher the potential targets of EBV miRNAs in this matching miRNA-mRNA dataset. In addition to the matching 25 EBV positive samples we downloaded 25 gastric adenocarcinoma EBV negative matching mRNA and miRNA sequencing samples from TCGA (Supplemental Table S1). The average EBV miRNA expression in the high group was 7615.75 TPM (median = 313.69 TPM) (Supplementary Fig. S1A). In order to predict the human genes that are targeted by EBV miRNAs in the high samples in comparison to those samples with no detected EBV miRNA expression we performed a differential mRNA expression analysis between EBV positive and EBV negative samples (Supplementary Fig. S1B and S1C). Since no EBV mRNA was expressed in the second set of samples, we included all the expressed EBV mRNAs in the EBV high group to identify targets of EBV miRNAs on the EBV genome (Supplementary Fig. S1C). We first modeled the miRNA-mRNA relationship using Gaussian, Poisson, negative binomial, zero inflated Poisson and the zero inflated negative binomial models available in the package. The Gaussian assumption yielded the smallest AIC, implying this was the most appropriate distribution for analysis (Fig. 1A, Supplementary Fig. S2A).

#### Epstein Barr virus miRNAs targeting human genes

**Fig. 2:**
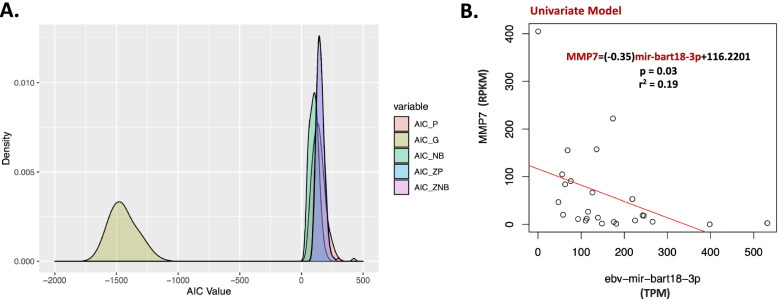
mirTarRnaSeq estimation of Epstein barr (EBV) miRNA targets on human 3′ UTR. **A.** Assessment of the miRNA-mRNA relationships across our sample cohort utilizing poisson (P), Gaussian (G), negative binomial (NB), zero inflated poisson (ZIP), and zero inflated negative binomial (ZNB) modes and comparing the AIC of these models. Note lower AICs generally report better performance. **B.** Univariate regression model estimation for MMP7 and ebv-mir-bart18-3p. Each dot represents a sample, y -axis denoted scaled (X10) TPM of miRNA expression and the x-axis represents the MMP7 mRNA RPKM

**Fig. 3:**
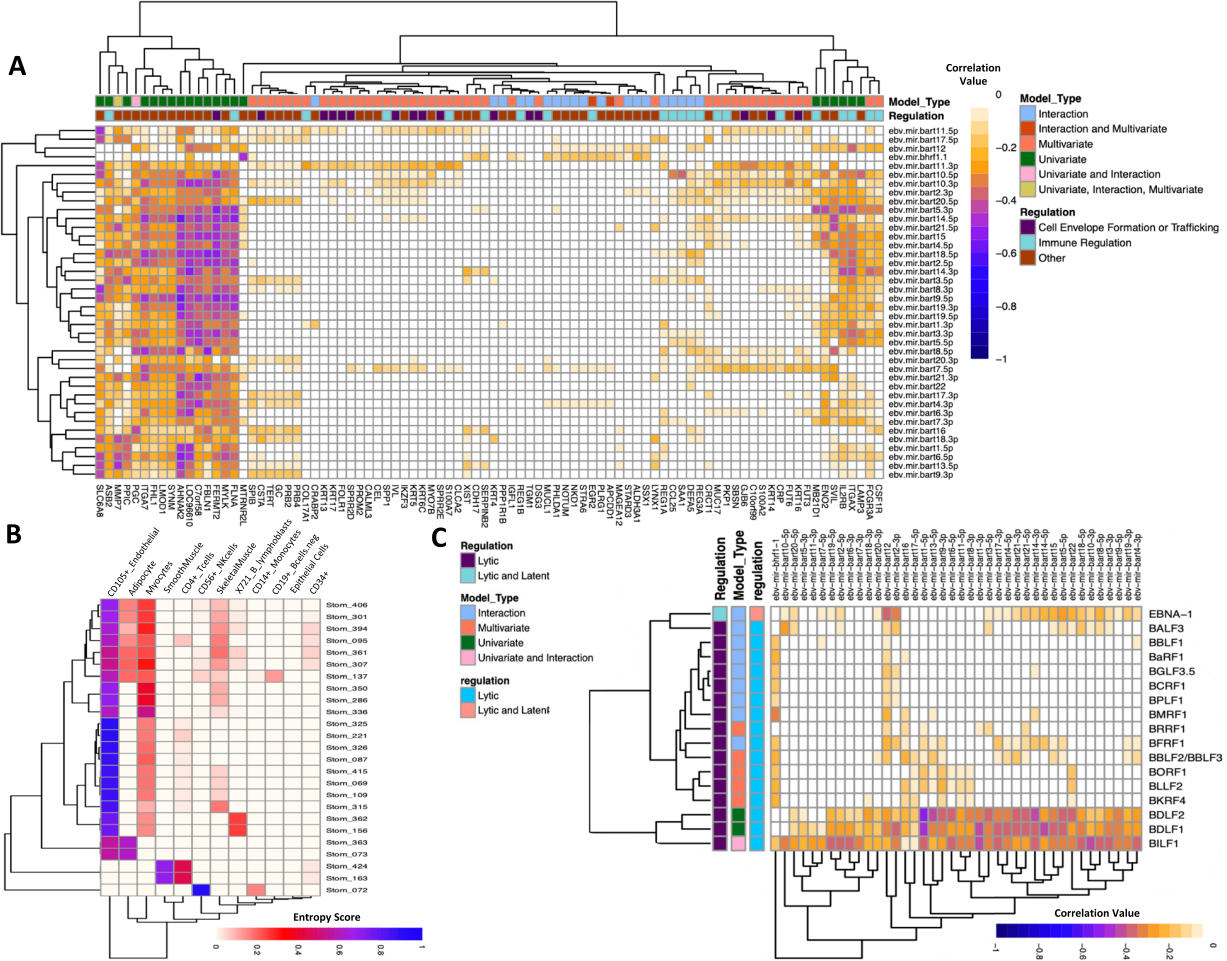
Targets of Epstein barr (EBV) miRNAs across human 3’UTR and EBV genome. **A.** Correlation heatmap for significant targets of EBV miRNAs on human 3’UTR. X-axis represents the human mRNA transcript names and the y-axis represents the viral miRNA names. Model type denotes the specific type of model which was used to make the prediction. Regulation annotation is obtained either through Reactome pathway prediction or specific literature on the gene in correlation with EBV or immune system regulation. Further details on the regulation can be found on Supplemental Tables S2 and S3. **B.** Heatmap of cell-type deconvolution for targets of EBV miRNAs on the human 3′ UTRs shown across the 25 samples. X-axis represents the sample names and Y-axis represent the cell type identified with genes targeted by miRNAs. **C.** Correlation heatmap for significant targets of EBV miRNAs across EBV genome. Model type denotes the specific type of model which was used to make the prediction. Regulation annotation is obtained through literature search. X-axis represents EBV mRNA names, and Y-axis represent EBV miRNA names

We applied *mirTarRnaSeq* to investigate if potential miRNA-mRNA interaction predicted by tools such as miRanda were differentially regulated. We observed 120 genes with evidence of EBV miRNA human target mRNA regulations (supplementary Table S3). Thirty-seven targets were differentially regulated in our univariate miRNA-mRNA analysis, 58 genes from a multivariate regulation analysis, and 32 from an interaction regulation analysis (Fig. 2A, supplementary Table S2). One gene, *MMP7*, a molecule previously shown to be regulated by EBV presence, was shown to be the target of EBV miRNAs through all three regression models (Fig. 2B, Supplementary Fig. S2B & S2C )[34]. The *PCG* gene was shown to have miRNA-mRNA regulation through both a univariate and interaction model while the *EGR2*, *CES1*, *APCDD1*, and *CRABP2* were regulated by both univariate and interaction models across the dataset. Of these 120 human genes, 88 were concurrently predicted by miRanda to be potential targets of these miRNAs as well (miRanda score > =140, N of potentially predicted targets by miRanda = 23,346 potential targets) (supplementary Table S2). Table 1 demonstrates the details of the results of each model with the number of potential miRNA and genes regulated predicted by each. We then estimated the *R* ^2^ value across the samples for miRNA-mRNA relationship to ensure the results of our predictions with another measure (Fig. 3A). We identified negative correlation for all univariate miRNA-mRNA models and at least one negative correlation for miRNA-mRNA multivariate and interaction models when run in a combination of two (Fig. 3A). Our univariate analysis identified multiple miRNAs targeting the same mRNA supporting the previous observation that more than one miRNA might be required for repression/downregulation of a gene [18]. Pathway analysis, with Reactome, revealed that targets of the genes are involved in the immune regulation (*n* = 22), cell envelope formation or trafficking (*n* = 16), or various other cellular functions (*n* = 50) (Fig. 3A and Supplemental Table S3 )[35]. We next performed human mRNA expression deconvolution to further investigate specific cell type transcripts that EBV miRNA target [30]. We found high levels of targeting on the CD105+ endothelial cells, monocyte, CD4+ T cells, NK cells, smooth muscle cells, CD19+ B cells and CD34+ cells across samples (Fig. 3B).

**Table 1:** Overall Model Results for EBV miRNAs Targeting Human and EBV Genomes. # represents the number of miRNAs or mRNAs. “_Human” denotes EBV miRNAs targeting human 3’UTR and “_EBV” emphasizes EBV miRNAs Targeting EBV genome. CoPredicted_miRanda defines those miRNA-mRNA regression models detected to have a significant relationship (< 0.05 adjusted *p* value) by mirTarRnaSeq as well as miRanda prediction

Model	#miRNAs	#mRNA	#CoPredicted_miRanda	% miRanda_Co-Prediction
**Univariate_Human**	37	37	28	62.16
**multivariate_Human**	19	58	16	81.03
**Interaction_Human**	32	32	29	71.88
**Univariate_EBV**	17	5	6	60
**multivariate_EBV**	19	11	6	45.45
**Interaction_EBV**	25	20	23	65

#### Epstein Barr virus miRNA targeting its own genes

In order to identify the targets of EBV miRNA on its own genome, we first modeled the miRNA-mRNA relationships and the Gaussian model again yielded the lowest estimated AIC (Supplementary Fig. S2A). We found 29 unique mRNAs predicted to be targeted by EBV miRNAs (Supplemental Table S4). Of these, 17 mRNAs were also predicted to be targets of EBV miRNAs by miRanda while 12 were uniquely identified by *mirTarRnaSeq* (Table 1). *BILF1* gene was the only gene which was predicted to be an miRNA target by both the univariate and the interaction model (Supplemental Table S4). Concordant with the results obtained from EBV miRNAs targeting of host mRNAs, most of the viral mRNAs identified as potential targets were targeted by other multiple EBV miRNAs (Fig. 3C). The one gene not induced by viral lytic activation was *EBNA1*, which is expressed at both lytic and latent phase. However, it is known that high levels of this transcript induce a lytic state hence downregulation of this gene is still consistent with repression of a persistent lytic state by EBV miRNAs to aid in viral latency [36]. This lytic to latent transition and regulation through EBV miRNAs has been previously reported, as EBVGC is known to go between latencies (I and II) expressing only EBERs in addition to *LMP2A* and miR-BARTs [37](Fig. 3C and Supplementary Fig. S2A, S2D and S2E).

### Part 2. *mirTarRnaSeq* time course analysis of SARS-CoV-2 infected human lung epithelial cells (correlation)

**Fig. 4:**
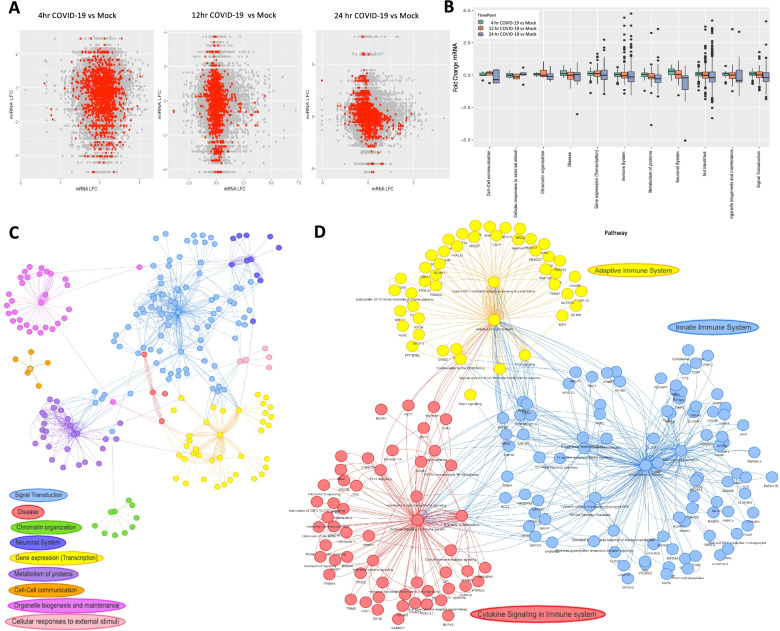
Predicted miRNA-mRNA correlations following SARS-CoV-2 infection of lung epithelial cells using mirTarRnaSeq. **A.** Distribution plot of miRNA and MRNA log fold changes (LFC) of SARS-CoV-2 infected across the three time points versus uninfected control cells. The red dots represent the correlations which are significant between the miRNAs and mRNA predicted by mirTarRnaSeq and miRanda (*p <* 0.05). **B.** Box plot of mean fold changes across three time SARS-CoV-2 infected versus uninfected control cells for all genes belonging to a specific pathway characterized by Reactome (*p <* 0.05). **C.** Network of targets and enriched pathways predicted to be correlated with miRNA expression predicted by both miRanda and mirTarRnaSeq significantly and Reactome respectively. Each node represents a gene, subpathway and pathway respectively, the edge distance is estimated by Barnes Hut simulation for gene-pathway connection. The color represents the pathways the targets belong to, predicted by Reactome (P mirTarRnaSeq and Reactome < 0.05) (Supplemetal_File 1). **D.** Network plot of all the targets of miRNAs involved in “Immune System”. Each node represents a gene, subpathway and pathway respectively, the edge distance is estimated by Barnes Hut simulation for gene-pathway connection. The color represents the pathway the targets belong to predicted by Reactome (Cytokine Signaling, innate and adaptive immunity)(Supplemetal_File 2)

Human mRNA targets of human miRNAs were predicted in triplicate Calu-3 lung epithelial cells collected 4 h, 12, and 24 h after infection with SARS-CoV-2 or uninfected control cells. DESeq2 was used to calculate differential expression for mRNA and miRNAs for SARS-CoV-2 infected versus uninfected control cells at each timepoint (Supplementary Fig. S3A,S3B,S3C,S3D,S3E and S3F). Across the three timepoints, 11,627 mRNAs and 687 miRNAs were included in the miRNA-mRNA correlation analysis, leading to 7,987,749 potential mRNA-miRNA interactions (Fig. 4A), including 37,519 with a miRanda interaction score greater than the 99th percentile of all miRanda interaction scores (miRanda score > 168). With a significance threshold of *p <* 0.05, 447,666 mRNA-miRNA correlations were significant, of which 2189 had a miRanda score > 168 (red dots in Fig. 4A). Non-significant interactions had a broader range of miRNA-mRNA correlation values than significant pairs (Supplementary Figure 4A). Reactome analysis of 1350 unique genes predicted to be regulated by miRNAs identified a complex network with 2416 nodes and 10,582 edges potentially regulated/involved with host miRNAs. Reactome pathway analysis of the differentially expressed genes identified 156 immune system genes with increased expression at 24 h after infection (Fig. 4B). Comparing those correlations with miRanda score above the 99th percentile (miRanda score > 168) to the entire input, there was enrichment for mRNAs involved in functions related to synaptic transmission and Fc-gamma receptor signaling involved in phagocytosis (Supplementary Figure 4B). When both input genes and selected genes are subset to those associated with the gene ontology term “immune response”, selected genes are enriched for functions related to Fc receptor signaling (FcRs) (Fig. 4C). A total of 7665 significant miRNA-mRNA interactions with a correlation of at least − 0.85 (Supplemental Table S5). Four hundred and forty-six of these interactions intersected with miRanda binding predictions. Reactome analysis of the 416 total interactions identified enrichment for biological functions including signal transduction, gene transcription, and metabolism of proteins (Fig. 4C). The use of both miRanda and TargetScan scores subset this list further, highlighting the potential to use multiple binding predictions in this package (Fig. 4D). Six miRNA-mRNA pairs had a miRanda score > 168, differential expression *p*-values < 0.05, and *mirTarRnaSeq* p-value < 0.05 (Table 2). Those pairings included *miR-93-5p* and *MAPK1*, a previously hypothesized regulatory pairing, and *miR-23c* and *ABL2,* and 5 of the 6 were also predicted as targets for miRNAs by TargetScan [14, 39–41]. When subsetted on the reactome term “Immune System”, functional enrichment was identified for cytokine signaling, innate immune system, and adaptive immune system (Fig. 4D).

**Table 2:** miRNA-mRNA Correlations Predicted by mirTarRnaSeq and Significantly Differentially Expressed in SARS-CoV-2 Infected vs Uninfected Controls Across 3 Time Points. Significant correlations between miRNA-mRNA for SARS-CoV-2 infected vs uninfected control cells differentially miRNA and mRNA expression (*P <* 0.05). Pink cell color signifies previously known miRNA-mRNA interactions (Tarbase v8), and green cell color represents known associated with endothelial damage due to SARS-CoV-2 infection [38]. Correlation value is the level of inverse correlation of miRNA and mRNA regulation estimated by mirTarRnaSeq observed in the dataset (− 1,0), where − 1 is at the most inverse correlation observed in the dataset). Target scan CSP represents context score percentile (between 1 and 100, where 100 is the highest score possible). mirTarRnaSeq *p* value calculation is described in the results section, the DE *p* value is estimated from differential expression analysis performed using DESeq2. NA represents a site not predicted as the target of miRNA by TargetScan

miRNA	Gene Symbol	Correlation Value	miRanda Score	TargetScan CSP	***mirTarRnaSeq p*** value	***p*** value mRNA DE	***p*** value miRNA DE
*hsa-miR-17-5p*	*SSH1*	−0.99	169	94	0.01807	0.001407784	0.00074288
*hsa-miR-93-5p*	*MAPK1*	−0.99	169	96	0.0005	0.038078634	0.001500608
*hsa-miR-93-5p*	*HOMER2*	−0.99	169	NA	0.00293	0.028214162	0.001500608
*hsa-miR-23c*	*ABL2*	−0.99	173	74	0.00136	0.027869177	0.0258363
*hsa-miR-374a-5p*	*LARP1*	−0.99	171	65	0.03374	0.01177344	4.25E-11
*hsa-miR-429*	*LONRF2*	−0.99	169	68	0.02127	0.018978845	1.01E-05

### Part 3. *mirTarRnaSeq* time course analysis of SARS-CoV-2 infected human lung epithelial cells for two time points (Interrelation)

**Fig. 5:**
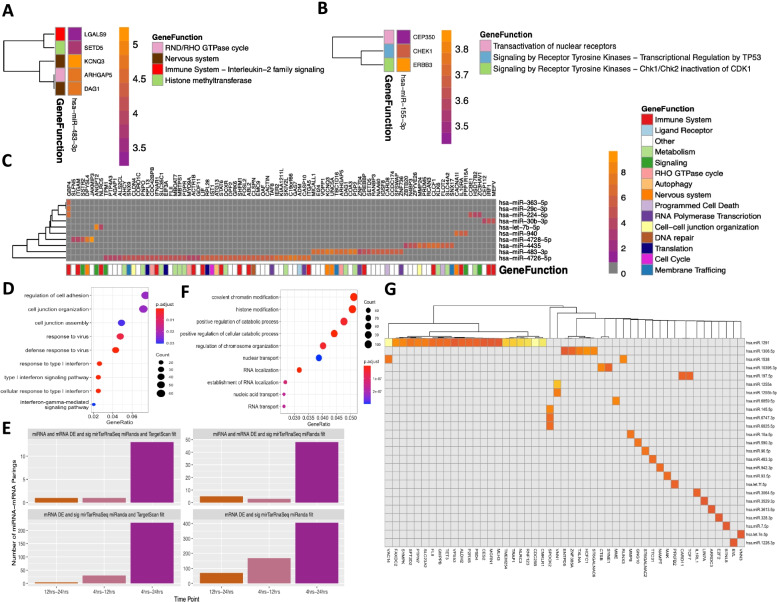
Predicted Targets of human miRNAs across time following SARS-CoV-2 infection of lung epithelial cells identified by mirTarRnaSeq through interrelation analysis. Interrelation heatmap representing significant (mirTarRnaSeq adjusted *p <* 0.1 and miranda score 169, for miRNA and mRNA differential expression (DeSEq2 adjusted *p <* 0.1) and mirTarRnaSeq predictions) miRNAs and mRNAs at **A** 4 to 12 hrs, CHEK1 is also a predicted target of hsa-miR-155-3p based on TargetScan algorithm. **B** 12-24 hours **C.** 4 vs 24 hrs heatmap of all significant miRNA-mRNA interrelations predicted by both mirTarRnaSeq and miRanda. The heatmap units are the absolute difference between miRNA and mRNA fold changes (FC); gray color represents no significant interrelation identified by mirTarRnaSeq. For all heatmaps, columns represent the significantly differentially expressed miRNAs, vs the mRNA targets shown in rows identified by mirTarRnaSeq. **D** GO enrichment analysis for all the targeted genes in part C which are targets of human miRNAs in 4-24 hrs time point interval. **E** Comparison of mirTarRnaSeq miRNA-mRNA interrelations analysis with miRanda and Target scan filtering step predictions. **F** Gene ontology enrichment for all mRNAs predicted to be miRNA targets by mirTarRnaSeq (any miRanda score). **G** Heatmap of all miRNA-mRNA interrelations predicted by mirTarRnaSeq and miRanda in patient blood after COVID-19 infection (*n* = 8, 4 with COVID-19, 4 without COVID-19). The heatmap units are the absolute difference between miRNA and mRNA fold changes (FC); gray color represents no significant interrelation identified by mirTarRnaSeq

In order to identify differences in miRNA-mRNA relationships between two timepoints, we calculated differential expression for mRNA and miRNAs for SARS-CoV-2 at 4 h versus (vs) 12 h, 4 h vs 24 h, and 12 h vs 24 h (Supplemental Fig. 5). Across the three intervals, a total of 11,721 mRNAs and 725 miRNAs were included for at least one time interval analysis. Using significance threshold of *p <* 0.01 and a miRanda interaction score greater than the 99th percentile of all scores (miRanda score > 168), there were 222 significant correlations at 4 h vs 12 h, 187 significant correlations at 4 h vs 24 h, and 498 at 12 h vs 24 h.. miR-155-3p was predicted to target 3 different mRNAs during the 12 h vs 24 h interval (Fig. 5A). During the 4 h vs 12 h interval, *miR-483-3p* was predicted to target 5 mRNAs (Fig. 5B) Over the 4 h vs 24 h interval, 10 miRNAs were predicted to target 94 unique mRNAs, 15 of which are identified by Reactome as having a function in the immune system (Fig. 5C). Three miRNAs were predicted to target *GBP4*, which is an interferon-stimulated gene. Treatment of SARS-CoV-2-infected lung epithelial cells with anti-inflammatory JAK-1/2 inhibitor Ruxolitinib led to decreased expression of *GBP4*, consistent with coregulation of *GBP4* mRNA by host miRNAs as a potential anti-inflammatory signal [42]. *miR-4726-5p* was predicted to target 45 mRNAs over that interval and all but three of the miRNAs were predicted to target more than one mRNA. We categorize the miRNA-mRNA interactions in four different terms: negative regulations, where the miRNA is significantly upregulated and mRNA is downregulated; inverse regulation, where the miRNA is significantly downregulated and the mRNA is upregulated; coregulation (increase), where both the miRNA and mRNA are upregulated; and coregulation (decrease), where both the miRNA and mRNA are downregulated. Significant miRNA-mRNA correlations which included an mRNA gene in the gene ontology term “immune response” were predominantly involved in interferon response and cytokine signaling, and the majority were inverse regulation (Table 3). Eighteen immune genes predicted to be targeted by miRNAs were differentially expressed at the 4 h vs 24 h interval, nine at the 4 h vs 12 h interval, and only *LAGLS9* was predicted to be targeted at the 12 h vs 24 h interval (Table 3). At the 4 h vs 24 h interval, 331 miRNAs were differentially expressed with an adjusted *p*-value < 0.05. At both the latter two intervals, as very few miRNA were differentially expressed: *miR-155-3p and miR-4485-3p* at 4 h vs. 12 h; *miR-12,136, miR-4284, miR-4463, miR-4485-3p*, *miR-483-3p*, and *miR-6891-5p* at 12 h vs. 24 h. *miR-483-3p* was predicted to target *CREBBP*, which has roles in both cytokine and interferon signaling (Supplemental Tables S6, S7 and S8).

**Table 3:** miRNA-mRNA Interrelation Involving Cytokine Immune Response Genes for 4-12 and 12-24 hours. Immune gene specific mirTarRnaSeq p-value significant predictions (adjusted *p <* 0.05) with above 169 miRanda binding prediction for part 2 (miRNA-mRNA correlation) with mRNA differentially significantly differentially expressed. Fold change (FC) adjusted *p* value (padj) are from DESeq2 predictions. Interactions with at least mRNA padj significance are shown. Immune_pathway prediction is through reactome pathway enrichment analysis. mirTarRnaSeq provides the option to choose the type of miRNA-mRNA relationship as demonstrated by Type_Putative_miRNA Regulation. Target scan CSP represents context score percentile (between 50 and 100, where 100 is the highest score possible)

Symbol	miRNA	log2FC_mRNA	padj_mRNA	log2FC_miRNA	padj_miRNA	Type_Putative_miRNA Regulation	TargetScan CSP	Time Point
*GBP4*	*hsa-miR-224-5p*	−6.875^a^	0	−0.628^a^	0.09	Coregulation (increase)	62	4-24
*IFNAR1*	*hsa-miR − 4726-5p*	0.414^a^	0.0915	5.17	0.28	Coregulation (increase)	72	4-24
*IL10RA*	*hsa-miR-940*	−4.67^a^	7.96E-17	1.119	0.41	Inverse Regulation	84	4-24
*IL10RA*	*hsa-miR-548d-3p*	-4.67^a^	7.96E-17	1.08	0.99	Inverse Regulation	77	4-24
*IST1*	*hsa-miR-4726-5p*	−0.306^a^	0.0478	5.17	0.18	Inverse Regulation	89	4-24
*ZC3HAV1*	*hsa-let-7i-3p*	−4.34	0	0.45^a^	0.06	Inverse Regulation	81	4-24
*NLRP3*	*hsa-miR-1226-5p*	−2.82	0.02	0.79	0.99	Inverse Regulation	47	4-24
*RBPJL*	*hsa-miR-1180-5p*	−6.8	0.07E-2	1.08	0.41	Inverse Regulation	71	4-24
*TNFSF13B*	*hsa-miR-30d-3p*	−5.96^a^	8.15E-20	−0.41	0.39	Coregulation (reduction)	99	4-24
*CD38*	*hsa-miR-548o-3p*	−5.08	2.84E-07	0.03	0.99	Inverse Regulation	80	4-24
*CD38*	*hsa-miR-3173-3p*	−5.08	2.84E−07	-0.69^a^	0.55	Coregulation (reduction)	98	4-24
*CD38*	*hsa-miR-939-5p*	−5.08	2.84E-07	0.82^a^	0.99	Inverse Regulation	88	4-24
*APO3*	*hsa-miR-6735 − 5p*	-5.41^a^	3.86E-44	−1.12^a^	0.99	Coregulation (reduction)	81	4-24
*ZC3HAV1*	*hsa-miR-589-3p*	−4.34	0	0.38^a^	0.99	Inverse Regulation	30	4-24
*HLA-DOB*	*hsa-miR-6886-3p*	−6.69	8.95E-16	0.79	0.99	Inverse Regulation	48	4-24
*CD40*	*hsa-miR-873-3p*	−2.77	7.19E-22	0.96	0.99	Inverse Regulation	73	4-24
*CSF1*	*hsa-miR-6763-5p*	−3.85	5.42E−42	0.16	0.99	Inverse Regulation	76	4-24
*TRAF1*	*hsa-miR-3605-5p*	-4.92^a^	2.19E-16	−0.47	0.99	Coregulation (reduction)	39	4-24
*LIF*	*hsa-miR-4726-5p*	−0.22	0.1	5.17	0.27	Inverse Regulation	68	4-24
*NFKBIA*	*hsa-miR − 378a-5p*	-3.95^a^	5.16E-251	0.3	0.99	Inverse Regulation	98	4-24
*IFIT2*	*hsa-miR-4436b-3p*	−7.01^a^	0	1.06	0.51	Inverse Regulation	84	4-24
*CXCL1*	*hsa-miR-4640-3p*	−4.14	8.34E-150	0.26	0.99	Inverse Regulation	61	4-24
*HLA-E*	*hsa-miR-6734-5p*	−1.21	0	3.22	0.28	Inverse Regulation	94	4-12
*IFI44L*	*hsa-miR-1262*	−2.46	8.05E-63	0.74	0.6	Inverse Regulation	79	4-12
*IFI44L*	*hsa-miR-330-5p*	−2.46	8.05E-63	−0.17^a^	0.6	Coregulation (reduction)	57	4-12
*NFKBIA*	*hsa-miR-378a-5p*	−3.16	2.27E-217	0.1	0.48	Inverse Regulation	98	4-12
*NLRC5*	*hsa-miR − 331 − 3p*	-3.95^a^	4.14E-98	0.27^a^	0.24	Inverse Regulation	58	4-12
*OAS1*	*hsa-miR-423-5p*	-3.13^a^	3.10E253	0.18^a^	0.01	Inverse Regulation	98	4-12
*PML*	*hsa-miR-7845-5p*	−2.6	2.06E-85	3.22	0.28	Inverse Regulation	65	4-12
*TAP2*	*hsa-miR-7113-3p*	−1.66	1.22E-55	2.44	0.51	Inverse Regulation	89	4-12
*TRIM22*	*hsa-miR-199a/b-3p*	−4.97	3.47E-269	1.54	0.36	Inverse Regulation	73/93	4-12
*TRIM22*	*hsa-miR-3617*	−4.97	3.47E-269	0.79	0.81	Inverse Regulation	94	4-12
*TRIM5*	*hsa-miR-30b-3p*	−2.45	1.62E-82	0.50	0.19	Inverse Regulation	86	4-12
*LGALS9*	*hsa-miR-483-3p*	1.027	5.75E-17	4.26^a^	8.36E-10	Coregulation (increase)	91	12-24
*IL7R*	*hsa-miR-6747-3p*	3.38^a^	1.05E-06	0.19^a^	0.99	Coregulation (increase)	98	12-24

^a^Indicates mRNA/miRNA that is also differentially expressed in blood from patients with COVID-19. ​

### Assessment of predicted mRNA-miRNA relationships during COVID-19 infection (interrelation)

To determine if miRNA-mRNA relationships observed in human lung epithelial cells were relevant to clinical COVID-19 infection, we repeated our analysis using paired mRNA and miRNA libraries which were prepared from blood samples of 4 patients with COVID-19 acute infection and 4 participants without COVID-19 infection (control). We performed the interrelation analysis (Part 3) using *mirTarRnaSeq*, with an adjusted p-value threshold of 0.05 and found 2871 mRNAs and 314 miRNAs with differential expression p-value based on COVID-19 status (Supplemental Fig. 6). Twenty-five miRNAs were predicted to target 49 mRNAs (Fig. 5G). *miR-1291* was predicted to target 22 different mRNAs. There was no functional enrichment among mRNAs predicted to be targets with miRanda score > 168, but among predicted targets with any miRanda score (range [140-189]), mRNAs were enriched for functions related to neutrophil response and catabolic state (Fig. 5F). Next, we identified shared miRNA-mRNA relationships obtained from the latter clinical COVID-19 infection versus control blood samples, and the lung epithelial COVID-19 infected cell experiment (Supplemental Table S9-11). Using a significance threshold of adjusted p-value < 0.1 and a miRanda interaction score greater than the 99th percentile of all scores (miRanda score > 168), there were 54 significant interrelations observed in both experiments (Supplemental Table S9). miRNA and mRNA relationships identified in both experiments include *IL7R*, a marker of naive T-cells which has been identified as a marker of T-cell trajectories after severe COVID-19 infection [43], was predicted to be targeted by *hsa-miR-6747-3p* in both lung epithelial cells and patient’s blood. *Hsa-miR-6735-5p* and its target *APOL3*, a gene downstream of tumor necrosis factor alpha and involved in cytokine signaling, showed interrelation in both datasets predicted by *mirTarRnaSeq* [44]. *LGALS9*, the gene encoding Galactin 9, was identified as a target in both experiments, and has been implicated in the severe cytokine response associated with COVID-19 [45]. *CREBBP*, a gene known to be altered in response to COVID-19 infection, and *hsa-miR-483-3p* were not only differentially expressed in patients with COVID-19 but also had an inverse interrelation using *mirTarRnaSeq* (adjusted *p* value = 0.03 and miRanda score 173 (range [140 -189]) concordant with the results obtained from the lung covid infection experiment [46].

Finally, to determine if the cytokine specific miRNA-mRNA relationships observed in human lung epithelial cells were relevant to clinical COVID-19 infection, we assessed shared patterns of differential miRNA and mRNA expression in the blood of these patients. Of the 33 unique cytokine related miRNA-mRNA pairs predicted during SARS-CoV-2 infection of human lung epithelial cells (Table 3 & Supplemental Table S9-11), 28 pairs had both the mRNA and miRNA differentially expressed in the blood during COVID-19 infection. Of those, 11 also had differential mRNA expression associated with COVID-19 infection while 10 had differential miRNA expression. Three of the 27 mRNA-miRNA pairs had differential expression of both the miRNA and mRNA: *hsa-miR-224-5p* and *GBP4*, *hsa-miR-331-3p* and *NLRC5*, and *hsa-miR-423-5p* and *OAS1* (Table 3, see asterisk)**.**

## Discussion

microRNAs are known to have important roles in post-transcriptional regulation of genes and in turn protein production across various eukaryotic and viral genomes. These regulatory functions are mainly dependent on base-pairing which influences the translation or statibility of the target mRNA molecule [6]. We demonstrated *mirTarRnaSeq*’s utility in identifying miRNA-mRNA relationships using publicly available datasets in TCGA (EBV gastric adenocarcinoma) and SRA (SARS-CoV-2 infected epithelial lung cell lines) and found evidence of various known and potentially novel miRNA-mRNA relationships. Multiple miRNA-mRNA relationships corroborated in a new dataset of blood acute COVID-19 infection as compared to control patients.

Given the prevalence and association of EBV with various autoimmunity, cancer and infectious diseases [47], we investigate the role of EBV miRNAs in regulating the host and its own genome’s transcription in 25 matching miRNA and mRNA stomach adenocarcinoma sequencing samples with high EBV infection levels [21] using *mirTarRNASeq*. Using various regression (univariate, multivariate and interaction/synergistic models) based options to identify miRNA-mRNA relationships available we unveiled previously identified and potentially novel EBV miRNA host mRNA interactions that could give insight into cell type specificity of miRNA-mRNA targeting in the host and EBV life cycle predictions. We identified multiple instances of several EBV miRNAs targeting a single mRNA, which could lead to better regulation of the target of interest as previously described. Notably, we find evidence for two novel instances of the EBV miRNA, *ebv-mir-bart14-3p* and *ebv-mir-bart5-3p*, targeting the interleukin 2 receptor subunit beta (*IL2RB*). *IL2RB* is well known to be a regulator of interleukin pathways and recently it has been reported that mutations in this gene result in susceptibility to EBV and CMV infections in addition to autoimmune disease [48]. We found evidence that a gene previously shown to associate with EBV infected gastric adenocarcinoma, *SPP1*, a member of the interferon gamma pathway, was targeted by two EBV miRNAs *ebv-mir-bart15* and *ebv-mir-bart5* [49]. Further, we report evidence for high levels of EBV miRNAs in CD105+ endothelial cells, monocytes, CD4+ T cells, NK cells, smooth muscle cells, CD1 + 9 B cells and CD34+ cells. Previous studies have reported deregulations of aforementioned pathways in EBV infections [50–55]. By evaluating the EBV miRNA host and viral mRNA interactions, there was strong evidence for a transition from viral lytic to latent cycle in these cells. A vast number of EBV lytic mRNAs potentially targeted by EBV miRNAs across this sample cohort conferring a lytic to latent transition in the majority of the samples (24/25). Five different EBV miRNAs were identified with three different regression models to target the *MMP7* gene (Supplemental Table S3). MMP1, is upregulated by the EBV proteins LMP1 and Zta and upregulation of *MMP1* has been shown to confer the invasive properties of EBV associated cancers [34]. As Zta is a lytic gene [56] and we observe an overall trend in lytic to latency in the stomach adenocarcinoma cells, we confirm that EBV miRNA are involved in maintaining EBV latency through regulation of the host and their own transcripts. Leveraging various regression methods (Part 1) provided in *mirTarRnaSeq* we found evidence for novel, potentially important relationships for EBV miRNAs and host and viral transcripts through various regression models, which warrant further in vitro and potentially other in vivo investigations.

Part 2 and Part 3 analyzed SARS-CoV-2 infection from two perspectives using time point miRNA-mRNA correlation analysis: viral effect at 3 time points after infection and viral effect across 3 time intervals, respectively. The analysis is agnostic to the specific times selected, so for some experiments it may be that more comparisons over longer periods may be beneficial. Part 2 allows for prediction of miRNA-mRNA correlations based on inversely correlated expressions in all included time points. Relationships predicted with high confidence (high score) by miRanda were enriched for strongly negative correlations in Part 2, supporting the plausibility of the miRNA-mRNA binding. Immune-relevant mRNAs that were predicted targets of miRNAs during SARS-CoV-2 infection were primarily those related to innate immunity and cytokines (Fig. 4). It has been argued that differences in the innate immune response are responsible for the heterogeneity of outcomes after SARS-CoV-2 infection [57]. Our analysis identified many potential miRNA regulators of cytokine signaling and endothelial response to inflammation following infection with SARS-CoV-2. Genes predicted to be targets include *MAPK1* and *ABL2*, both of which have been implicated in the pathogenic host inflammatory response to SARS-CoV-2 disease [38, 58]. *MAPK1* is also a predicted target of SARS-CoV-2 viral miRNAs [59]. Since miRNA mimics can be delivered therapeutically, further research to establish the clinical relevance of *miR-93-5p* and *miR-23c* is warranted [60]. *mirTarRnaSeq* is only able to perform pairwise miRNA-mRNA interaction analyses for data collected longitudinally and is not able to perform spline based longitudinal modeling.

In contrast, Part 3 identified miRNA-mRNA interrelation by comparing the observed distribution of paired expression changes across individual time intervals to background expectation. This analysis provides temporal specificity to miRNA-mRNA predictions which could be useful for assessing relevance to cellular phenotypes or stages of disease. In the 24 h after SARS-CoV-2 infection there was significant enrichment for miRNA targeting of mRNAs involved in cytokine, interferon and interleukin signaling in lung epithelial cells (Table 3). Clinical relevance of the predicted relationships was further supported by demonstration of differential expression in blood of patients with COVID-19 among 3 of the 27 miRNA-mRNA pairs predicted in the cell culture experiments. High levels of cytokines have been detected from airway and blood samples of patients with COVID-19 and drive the cytokine storm of severe COVID-19 [61–64]. As with other hyperinflammatory disorders, inflammatory mediators such as interleukin-1 and interleukin-6 have been therapeutic targets in COVID-19 clinical trials with varying success [65, 66]. Characterizing the miRNAs which mediate host hyperinflammatory response to SARS-CoV-2 could be useful for prognostication, and potentially as adjunctive therapeutic targets.

We also utilized the interrelation model (Part 3) of *mirTarRnaSeq* to characterize miRNA and mRNA relationships in blood from patients with COVID-19. Overall, 36 mRNA-miRNA relationships found in both lung epithelial cells and patient blood datasets (Supplemental Table S9). Some of these miRNAs are potentially relevant to the host response to SARS-CoV-2, including *hsa-miR-6747-3p*, *hsa-miR-483-3p*, *miR-4726-5p* and *miR-4728-5p*. Replication of these analyses in a dataset that includes a pre-infection sample as baseline could improve sensitivity to identify potential therapeutic roles for miRNAs in COVID-19 infection. Further, modeling in Part 3 allows detection of a greater range of regulatory interactions including coeregulation, which would not be detected by Part 2. *miR-4726-5p* was predicted to have 5 co-regulatory interactions with immune genes, where both *miR-4726-5p* and its target mRNA had decreased expression following SARS-CoV-2 infection. Modulation of these individual miRNAs in COVID-19 models could identify key regulators that mediate these miRNA-mRNA relationships. As many previous models of miRNA-mRNA interactions selected only inverse regulatory interactions, new associations may be uncovered using this more flexible analysis.

In summary, *mirTarRnaSeq* implements statistical tests for identifying miRNA-mRNA relationships within high throughput datasets. *mirTarRnaSeq* can investigate these relationships in multiple eukaryotic and viral organisms and is not restricted to the analysis of human miRNAs. *mirTarRnaSeq* is freely available on a well maintained platform, Bioconductor, which provides easy access and clear vignettes and support for users of the package. With the current progress in RNA research, development of packages such as *mirTaraRnaSeq* are crucial to comprehend the extent of roles of non-coding RNAs in various organisms.

## Supplementary Information

**Additional file 1.**

**Additional file 2.**

**Additional file 3.**

**Additional file 4.**

**Additional file 5.**

**Additional file 6.**

**Additional file 7.**

**Additional file 8.**

**Additional file 9.**

## Data Availability

All data used for the analysis is available either in The Cancer Genome Atlas (TCGA, http://cancergenome.nih.gov/) accessed through the Cancer Genomic Hub [20] or Gene Expression Omnibus at NCBI (https://www.ncbi.nlm.nih.gov/geo). All pre-processed organism specific miRanda files are publicly available in Zenodo under (Version mirTarRnaSeq Version 1.2.2 10.5281/zenodo.4898541) and can be downloaded directly using *miRTarRnaSeq* package [67]. All test files for the package and EBV and human lung epithelial cell gene expression files are available on Zenodo, (DOI 10.5281/zenodo.5234277 and DOI 10.5281/zenodo.5348265, respectively**)**. Our COVID-19 fastq files can be accessed through SRA, Submission ID: SUB10649067 and under BioProject ID: PRJNA779882.

## Abbreviations

AIC: Akaike information criterion
AGO: Argonaut
circRNAs: Circular RNAs
CPM: Counts per million
Db: Database
DE: Differential expression
EBV: Epstein Barr Virus
EBVGC: Epstein Barr virus associated gastric carcinoma
FC: Fold change
HCMV) (NC_00627: Human cytomegalovirus
IL: Interleukin
KSHV: Kaposi sarcoma herpes virus
miRNA: MicroRNA
mRNA: Messenger RNA
ncRNA: Non-coding RNA
QC: Quality-checked
RPKM: Reads per kilobase of transcript per million mapped reads
Seq: Sequencing
SARS-Cov-2/COVID-19: SEVERE acute respiratory syndrome coronavirus 2
STAD: Stomach adenocarcinoma
TCGA: The Cancer Genome Atlas
TPM: Transcripts per million
Vs: Versus
