# Supplementary figures and images for: mirTarRnaSeq: An R/Bioconductor Statistical Package for miRNA-mRNA Target Identification and Interaction Analysis
Source: BMC Genomics. 2022 Jun 13;23:439. doi: 10.1186/s12864-022-08558-w (PMC9191533; doi:10.1186/s12864-022-08558-w)

# Supplemental Figure S1.

A

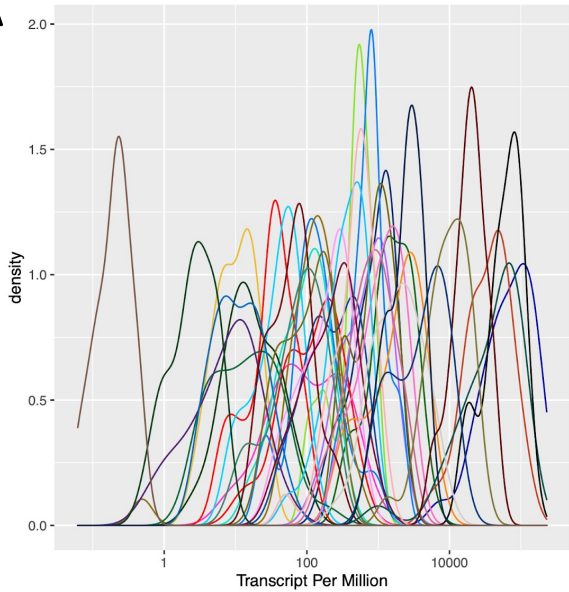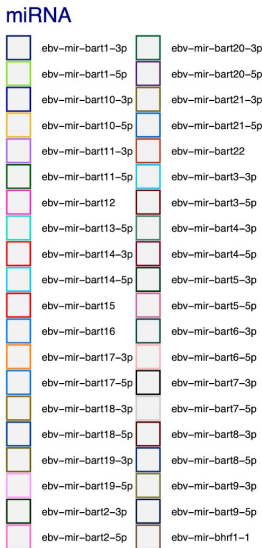

B

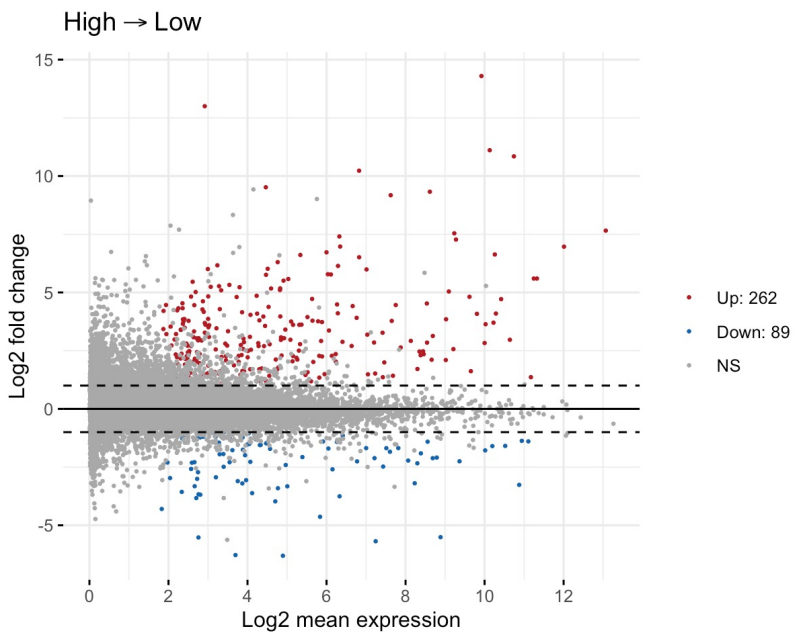

C

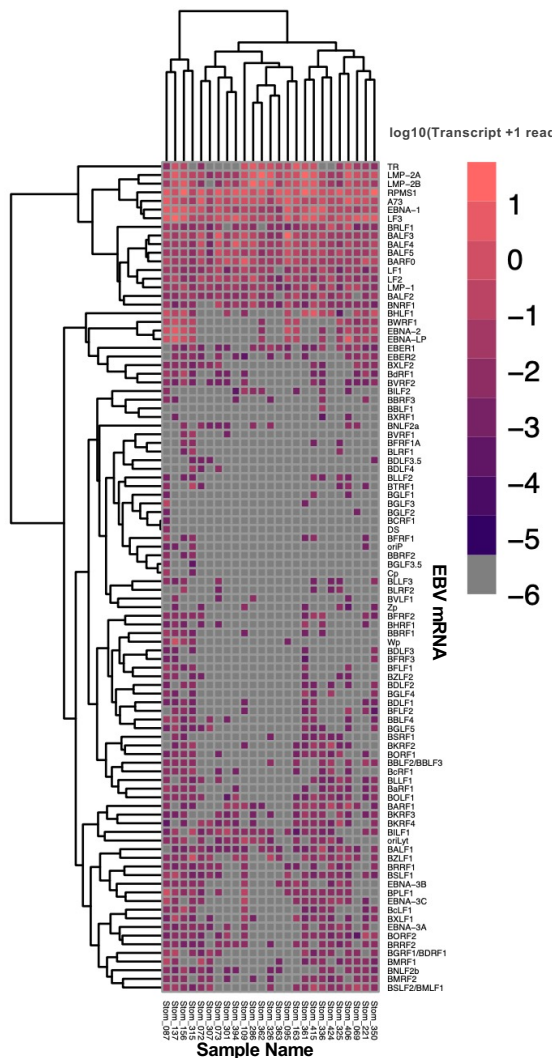

Supplement: Supplementary file 2 — Additional file 2. [file 12864_2022_8558_MOESM2_ESM.pdf]

Supplemental Figure S3

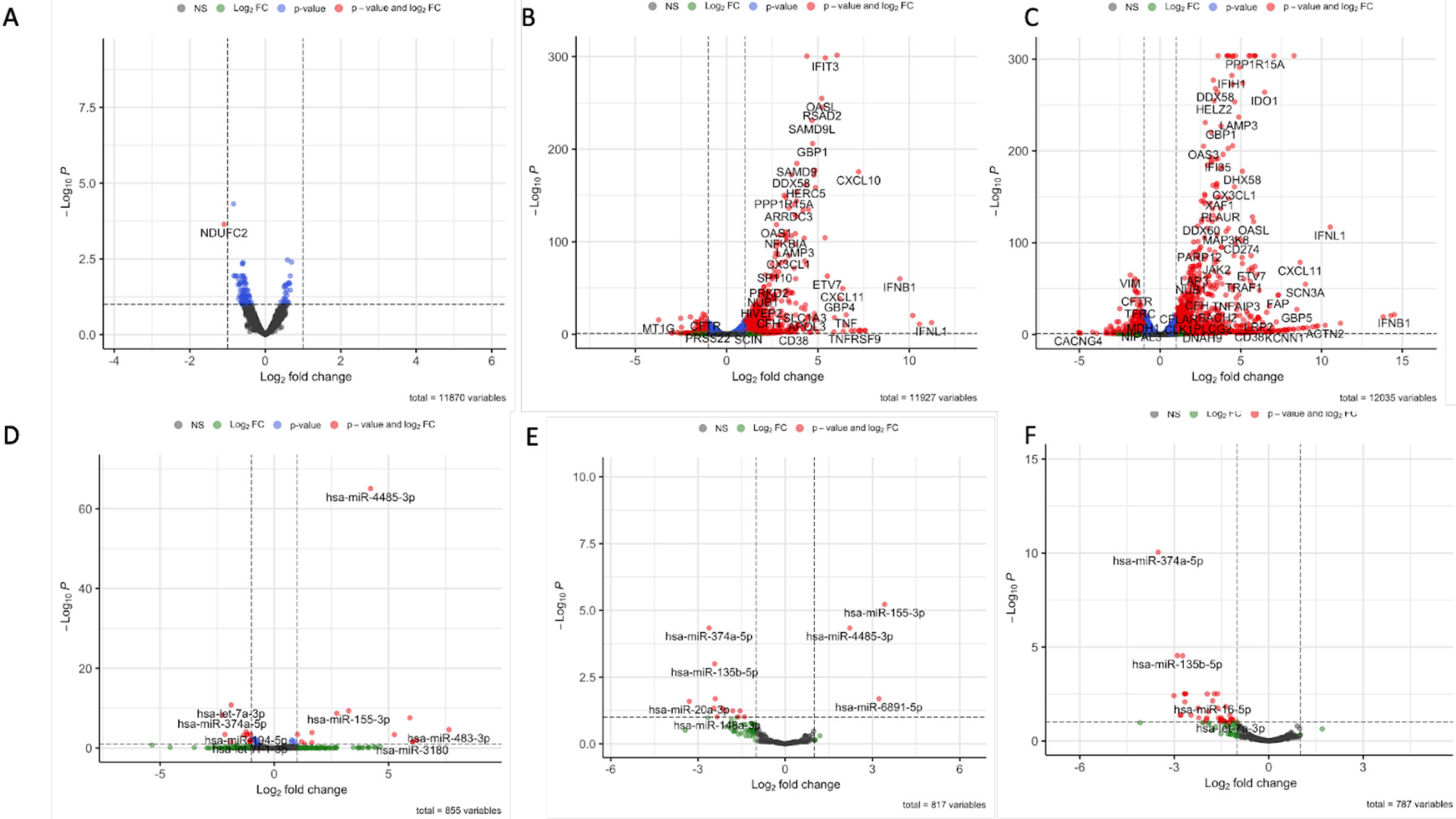

Supplement: Supplementary file 4 — Additional file 4. [file 12864_2022_8558_MOESM4_ESM.pdf]

# Supplemental Figure S4

A

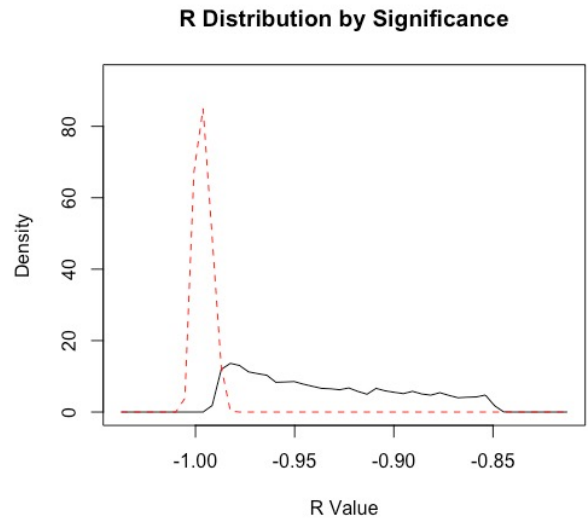

B

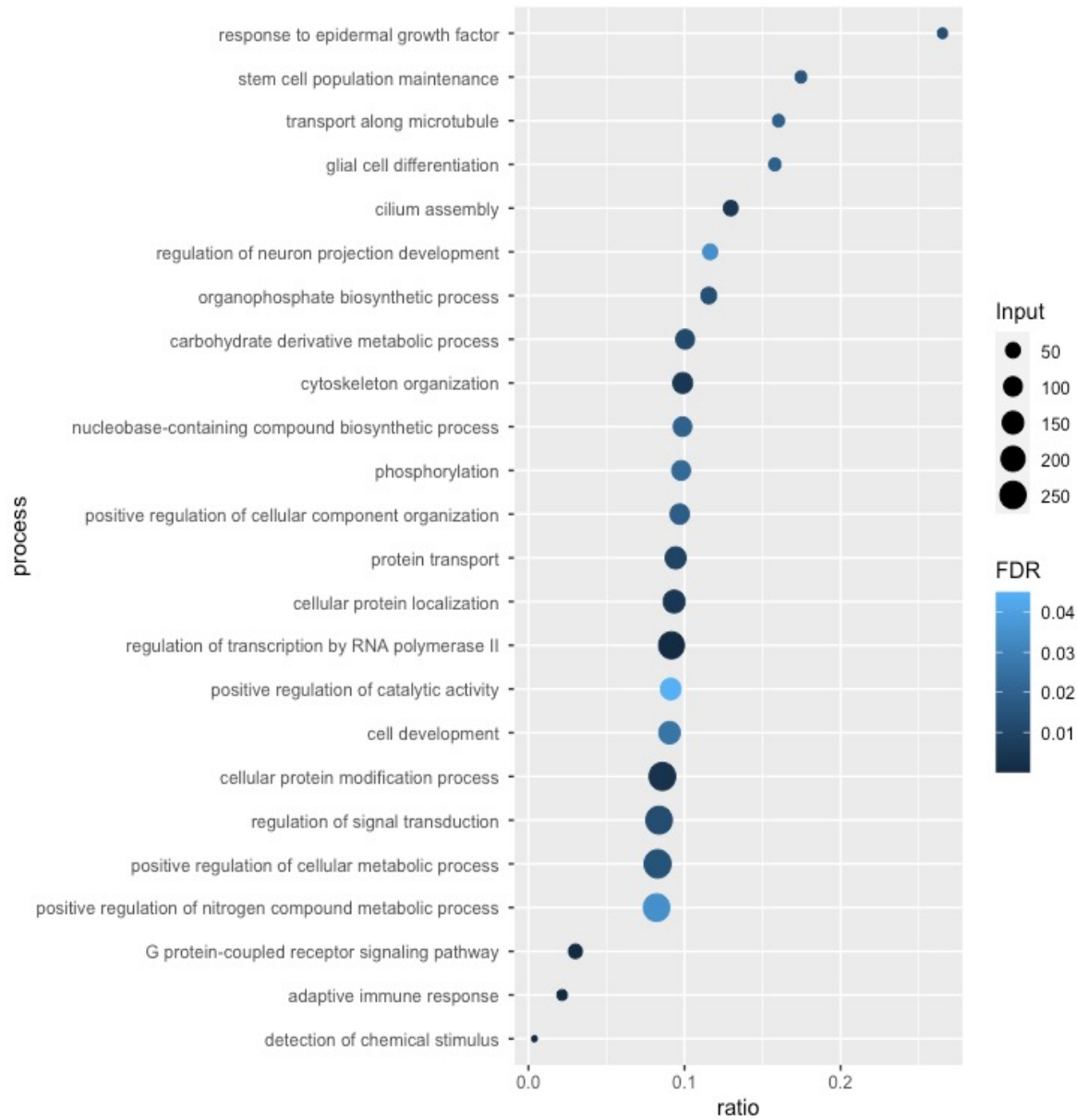

Supplement: Supplementary file 5 — Additional file 5. [file 12864_2022_8558_MOESM5_ESM.pdf]

# Supplemental Figure S5

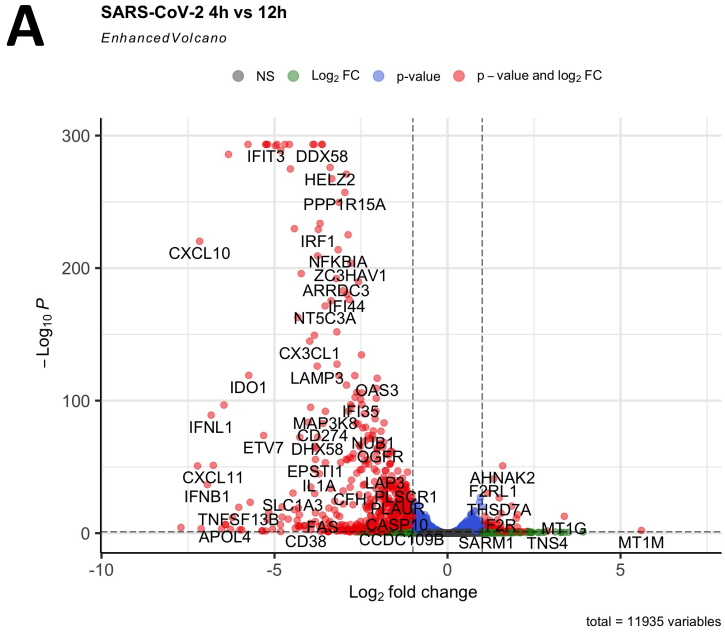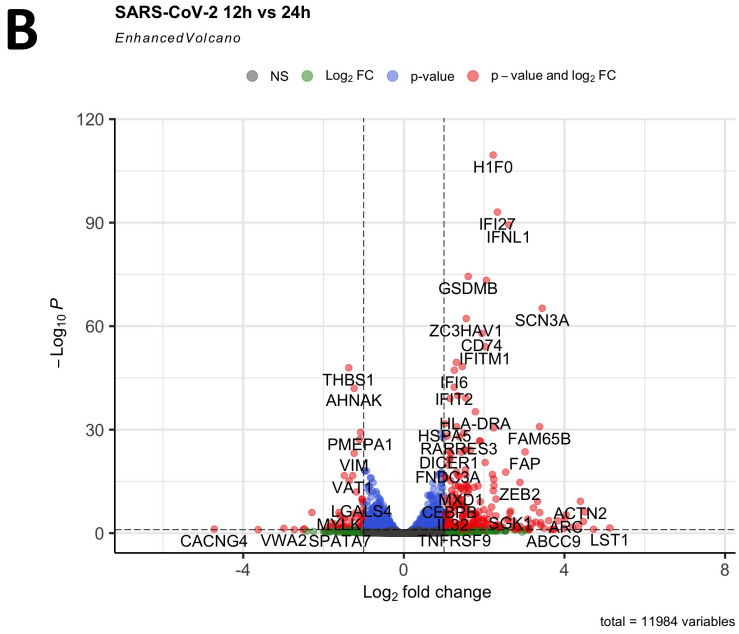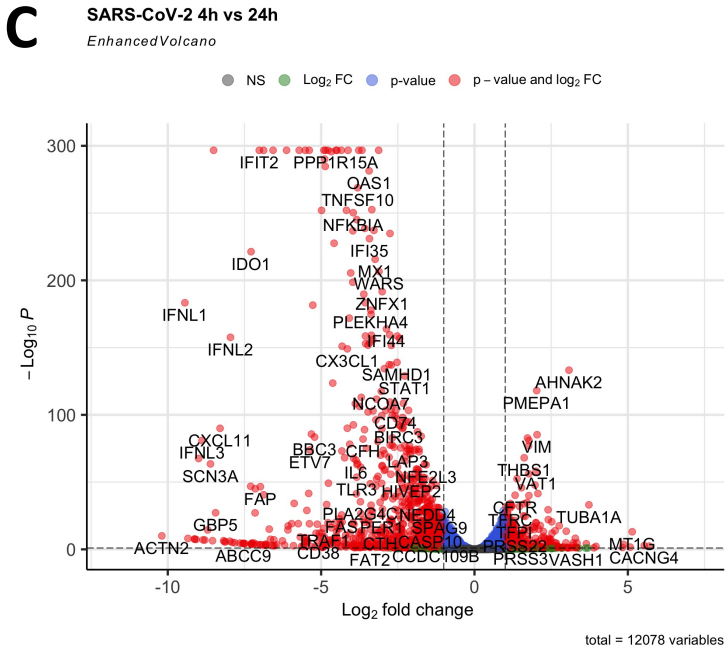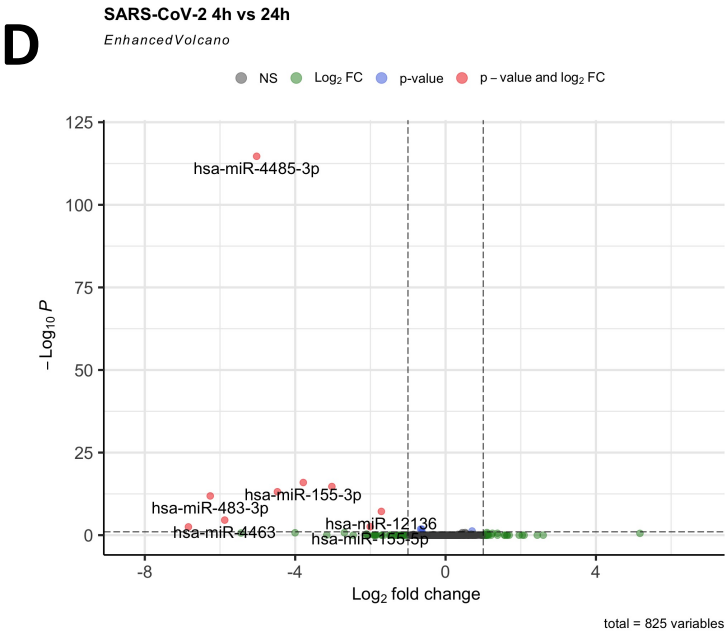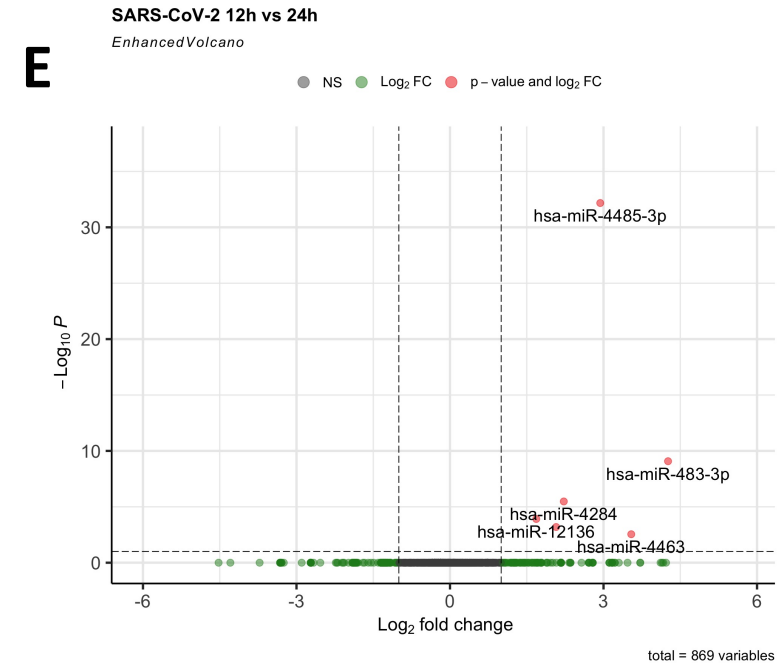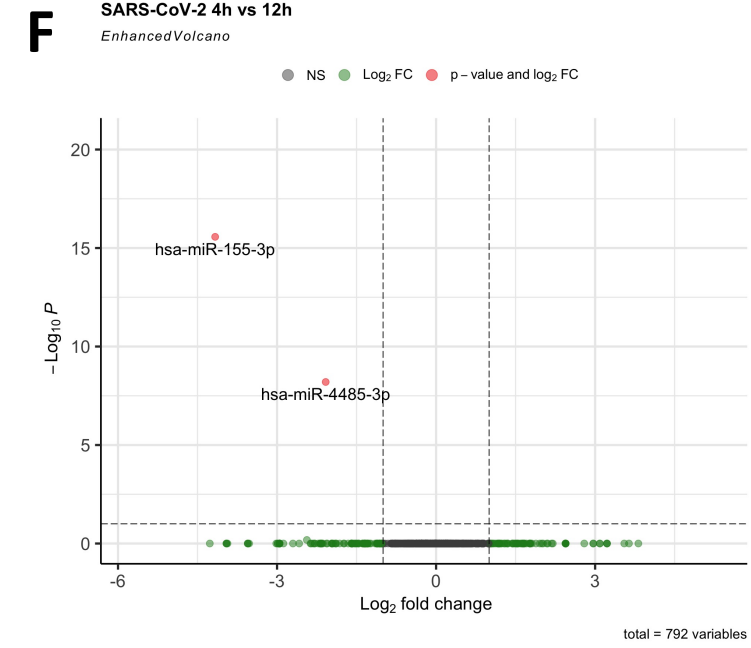

Supplement: Supplementary file 6 — Additional file 6. [file 12864_2022_8558_MOESM6_ESM.pdf]

# Supplemental Figure S6

A

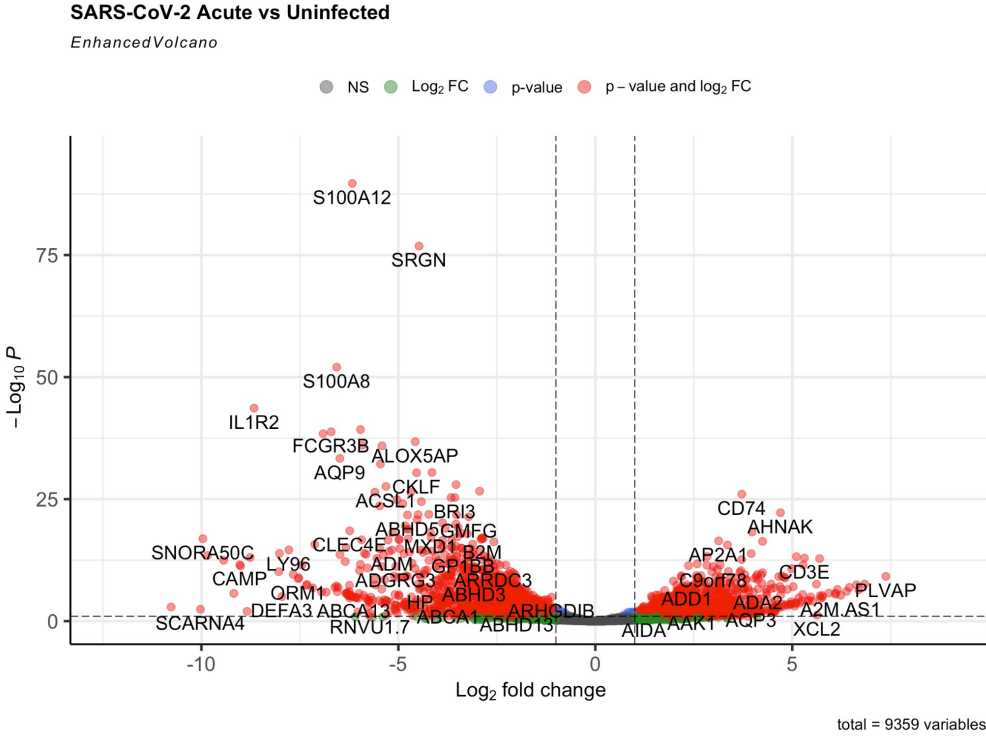

B

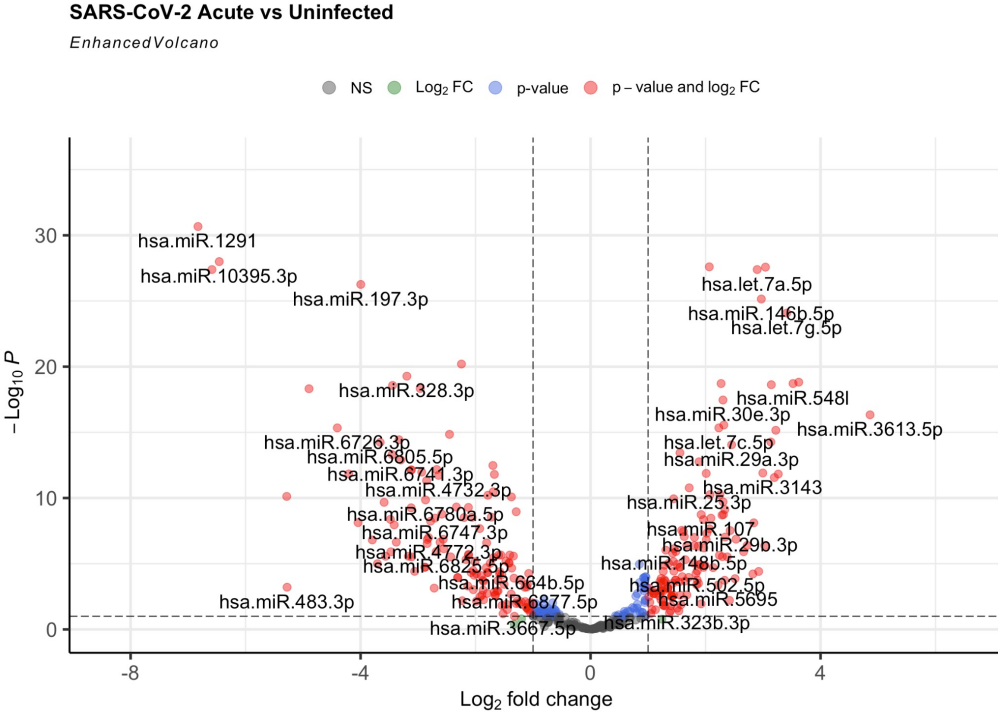

Supplement: Supplementary file 7 — Additional file 7. [file 12864_2022_8558_MOESM7_ESM.pdf]
