# Supplementary material for: mirTarRnaSeq: An R/Bioconductor Statistical Package for miRNA-mRNA Target Identification and Interaction Analysis
Source: BMC Genomics. 2022 Jun 13;23:439. doi: 10.1186/s12864-022-08558-w (PMC9191533; doi:10.1186/s12864-022-08558-w)

Supplemental Figure S2

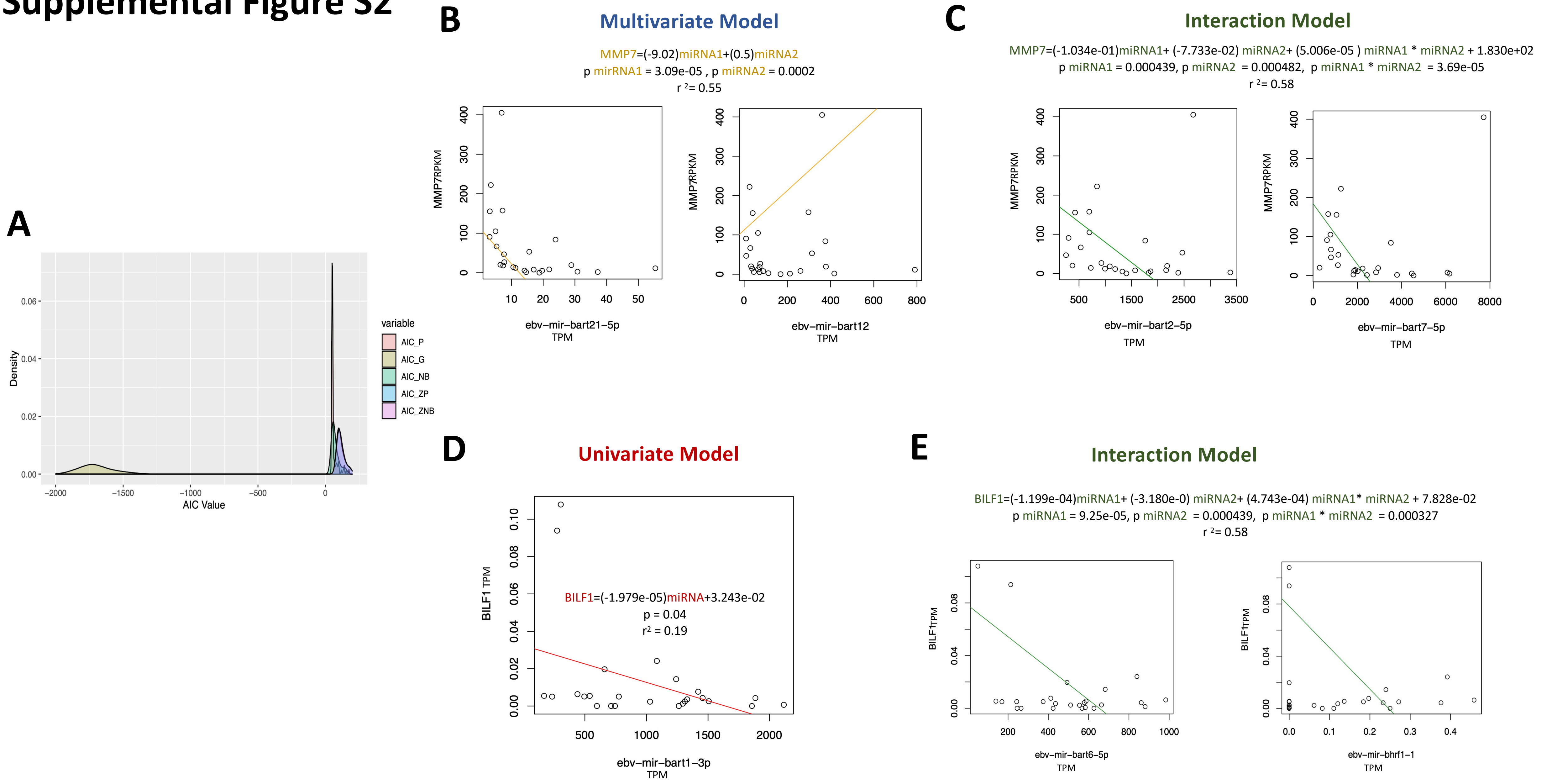

Supplement: Supplementary file 3 — Additional file 3. [file 12864_2022_8558_MOESM3_ESM.pdf]
